# Clinical Prediction Model for Unsuccessful Left Bundle Branch Area Pacing

**DOI:** 10.1111/jce.70339

**Published:** 2026-04-11

**Authors:** Yousaku Okubo, Junji Maeda, Shogo Miyamoto, Hiroshi Oe, Naoki Ishibashi, Shunsuke Ishida, Takumi Sakai, Naoto Oguri, Motoki Furutani, Shunsuke Miyauchi, Sho Okamura, Takehito Tokuyama, Noboru Oda, Yukiko Nakano

**Affiliations:** ^1^ Department of Cardiovascular Medicine Hiroshima University Graduate School of Biomedical and Health Sciences Hiroshima Japan

**Keywords:** conduction system pacing, left bundle branch area pacing, machine learning, nomogram, risk prediction model

## Abstract

**Introduction:**

Left bundle branch area pacing (LBBAP) has rapidly become a preferred form of cardiac physiologic pacing; however, predictors of unsuccessful implantation remain unclear.

**Methods and Results:**

Between April 2020 and March 2024, LBBAP was attempted in 145 patients. Procedural success was defined by ≥ 1 of the following: output‐dependent QRS morphology changes, V6 R‐wave peak time (RWPT) < 75 ms, V6–V1 interpeak interval ≥ 44 ms, or concordance between intrinsic and paced V6 RWPT derived from left bundle potentials. Baseline clinical, electrocardiographic, and echocardiographic variables were compared between success (*n* = 126, 86.9%) and failure (*n* = 19, 13.1%) groups. Multivariable logistic regression using Firth's penalized likelihood identified nonspecific intraventricular conduction delay (NIVCD) (odds ratio [OR] = 4.34; *p* = 0.02), interventricular septal thickness (IVSd) (OR = 1.39 per mm; *p* = 0.03), and right atrial area (RAA) (OR = 1.12 per cm^2^; *p* = 0.01) as independent predictors of unsuccessful LBBAP. A model integrating these variables demonstrated strong discrimination (area under the curve [AUC] = 0.84), which was confirmed by bootstrap validation (AUC = 0.85; 95% confidence interval = 0.76–0.93). A nomogram was constructed to enable individualized risk estimation.

**Conclusion:**

NIVCD, septal hypertrophy, and right atrial enlargement are independent predictors of LBBAP failure. The resulting validated prediction model and nomogram provide individualized pre‐procedural risk estimation and may assist in optimizing patient selection and procedural strategy.

## Graphical abstract

A clinical prediction model based on NIVCD, interventricular septal thickness, and right atrial area enables individualized estimation of unsuccessful LBBAP and may help guide procedural strategy.

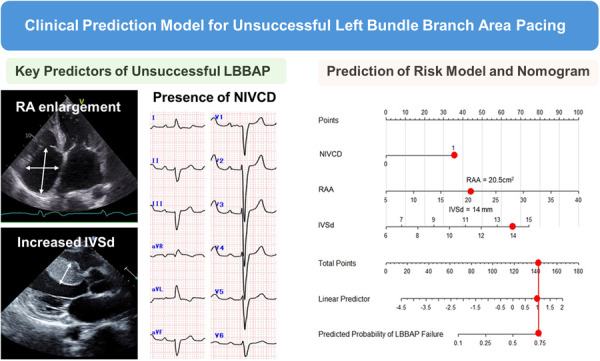

**Abbreviations**
IVSd: interventricular septal thickness
LBBAP: left bundle branch area pacing
LVDd: left ventricular end‐diastolic diameter
NIVCD: nonspecific intraventricular delay
RAA: right atrial area
RWPT: R‐wave peak time

## 1 Introduction

Left bundle branch area pacing (LBBAP) is a pacing method that maintains physiological ventricular activation by capturing the conduction system around the left bundle branch [1, 2, 3, 4]. It has become one of the most widely adopted forms of conduction system pacing because of its high procedural success rate and stable electrical parameters. Reported success rates typically range from 82% to 93%, highlighting its feasibility across diverse patient populations [5, 6, 7, 8, 9, 10, 11]. The electrocardiographic criteria used to define left bundle branch capture in this study were based on contemporary expert consensus recommendations. Although these criteria are widely adopted in clinical practice, definitive prospective data demonstrating superior long‐term outcomes compared with deep septal myocardial pacing remain limited. Nevertheless, unsuccessful implantation still occurs in a subset of patients, suggesting that patient‐specific anatomical or electrical factors may significantly influence procedural outcomes. Several characteristics have been associated with technical difficulty during LBBAP implantation, including left ventricular (LV) dilation, nonspecific intraventricular conduction delay, heart failure, indication for cardiac resynchronization therapy, baseline QRS prolongation, severe tricuspid regurgitation, and increased interventricular septal thickness, such as in hypertrophic cardiomyopathy [11, 12, 13]. However, prior studies have only identified associations rather than providing a method to estimate the probability of implantation failure for individual patients. Consequently, clinicians still lack a practical tool to anticipate difficult cases and adjust procedural strategy accordingly.

To address this unmet need, we conducted a retrospective cohort study of patients with atrioventricular block undergoing attempted LBBAP. Our objectives were to identify independent predictors of unsuccessful implantation and to develop an individualized prediction model with machine learning–based validation. A reliable pre‐procedural risk model may help guide device selection, inform procedural planning, and support risk adjustment in future evaluations of conduction system pacing technologies.

## 2 Methods

### 2.1 Study Design and Patient Population

**Figure 1:**
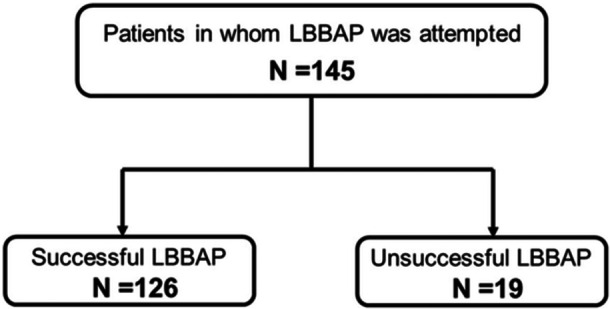
Flow diagram of the study cohort. A total of 145 patients underwent attempted left bundle branch area pacing (LBBAP) and were included in the analysis. LBBAP was successful in 126 patients and unsuccessful in 19 patients.

This single‐center retrospective study included 145 consecutive patients in whom LBBAP was attempted at Hiroshima University Hospital between April 2020 and March 2024. Patients with an indication for implantable cardioverter‐defibrillator (ICD), cardiac resynchronization therapy (CRT), or leadless pacemaker implantation were not included in this analysis. Patients in whom right ventricular pacing was selected due to an anticipated pacing burden < 20% were also excluded (Figure 1). The study was approved by the Institutional Ethics Committee of the Graduate School of Biomedical Science at Hiroshima University and conducted in accordance with the tenets of the Declaration of Helsinki. The institutional ethics committee determined that written informed consent was not required due to the retrospective observational nature of the study.

### 2.2 LBBAP Implantation Technique

The implantation technique for LBBAP in this study was performed according to the procedural principles described by Huang et al. in their beginner's guide to permanent LBBAP [14]. All procedures were performed using a lumen‐less lead (SelectSecure 3830 lead, Medtronic, Minneapolis, MN, USA) delivered through a fixed‐curve C315 His sheath (Medtronic). The delivery sheath was introduced into the right ventricle (RV) over a guidewire via the axillary vein. Although left‐sided implantation was the default approach, right‐sided implantation was pre‐decided in some patients due to venous access considerations and was not performed as a bailout strategy after unsuccessful left‐sided attempts. For LBBAP attempts, the His bundle region was first mapped to establish an anatomic reference. The sheath was then advanced 1.5–2.0 cm toward the RV apex from the His position and gently rotated counterclockwise to obtain perpendicular contact with the interventricular septum. Unipolar pacing was performed to assess paced‐QRS morphology. Lead deployment was initiated at sites demonstrating a characteristic “W” pattern with a notch in lead V1. During lead advancement, intermittent assessment of paced‐QRS morphology, lead impedance, and contrast angiography in the left anterior oblique (LAO) 30° projection was performed to evaluate the depth of lead penetration within the septum. A sudden decrease in unipolar impedance (> 200 Ω) or an absolute impedance < 500 Ω was considered suggestive of LV cavity penetration, prompting repositioning of the lead. If a successful LBBAP was not achieved after several attempts, LBBAP was abandoned, and RV pacing was performed based on operator preference. If successful, LBBAP could not be achieved despite multiple lead rotations and repeated septal advancement attempts; the procedure was discontinued at the operator's discretion. Attempts were abandoned when further advancement was judged unlikely to succeed due to marked septal resistance, inability to achieve deeper septal penetration, repeated failure to meet predefined electrophysiologic criteria, or concern for septal perforation. In such cases, right ventricular septal pacing was selected as an alternative strategy. All procedures were performed by experienced operators, each of whom had performed more than 50 LBBAP implantations prior to study initiation. Previous studies have suggested that procedural success improves after the initial learning phase of approximately 50 cases [15]. Therefore, the present cohort reflects procedures performed beyond the early learning curve.

### 2.3 Definition of Successful LBBAP

The electrophysiological criteria for successful LBBAP were based on previously published recommendations and expert consensus [16, 17, 18, 19]. LBBAP was considered successful when the pacing lead was securely embedded within the interventricular septum and when at least one of the following conditions was demonstrated: (1) an output‐dependent transition in QRS morphology assessed during unipolar pacing, (2) a V6 R‐wave peak time (RWPT) of less than 75 ms, (3) a V6–V1 interpeak interval of 44 ms or greater, or (4) concordance between the intrinsic and paced V6 RWPT derived from recorded left bundle potentials.

Unsuccessful LBBAP was defined as failure to demonstrate definitive left bundle branch capture according to these predefined criteria. Cases in which the pacing lead was advanced into the deep interventricular septum but did not fulfill ECG‐based criteria for left bundle capture were also classified as unsuccessful.

### 2.4 Baseline Electrocardiographic and Echocardiographic Measurements

Baseline electrocardiographic data, physical examination findings, echocardiographic measurements, and routine laboratory values were obtained at the time of hospital admission. Twelve‐lead electrocardiograms (ECGs) were recorded at a paper speed of 25 mm/s and a voltage of 10 mm/mV. QRS duration was measured as the longest value among all leads. Nonspecific intraventricular conduction delay (NIVCD) was defined as a QRS duration ≥ 120 ms that did not fulfill established electrocardiographic criteria for complete left bundle branch block or right bundle branch block according to standard guideline definitions [20]. The V6 RWPT was measured from the onset of the QRS complex to the peak of the R wave in lead V6. The V6–V1 interpeak interval was defined as the difference between the peak of the R wave in lead V6 and that in lead V1, measured on the same beat. Comprehensive transthoracic echocardiography was performed before device implantation according to the recommendations of the American Society of Echocardiography [21]. Interventricular septal thickness (IVSd) and posterior wall thickness were measured in the parasternal long‐axis view using M‐mode. Right atrial area (RAA) was quantified by planimetry in the apical four‐chamber view at end‐systole. Left ventricular ejection fraction (LVEF) was calculated using the modified Simpson biplane method. Chamber dimensions, valvular regurgitation severity, and estimated pulmonary artery systolic pressure were also assessed as part of the standard echocardiographic protocol.

### 2.5 Statistical Analysis

Continuous variables were expressed as mean ± standard deviation or median with interquartile range, depending on their distribution, and were compared between groups using the Mann–Whitney *U*‐test. Categorical variables were summarized as frequencies and percentages and compared using the *χ* ^2^ test or Fisher's exact test, as appropriate. Variables with *p* < 0.10 in univariable analysis, along with clinically relevant variables, were entered into a multivariable logistic regression model using Firth's penalized likelihood method to identify independent predictors of unsuccessful LBBAP. Adjusted odds ratios (ORs) with 95% confidence intervals (CIs) were reported. A receiver operating characteristic (ROC) curve was generated using the linear predictor derived from the final multivariable Firth logistic regression model to evaluate the model's discriminative performance. Model discrimination was assessed using the area under the receiver operating characteristic curve (AUC). Calibration was evaluated both visually using calibration plots and numerically by the calibration slope. Internal validation was performed using 1000 bootstrap resamples to obtain optimism‐corrected performance estimates. A nomogram was subsequently constructed based on the final multivariable logistic regression model. All statistical analyses were conducted using the rms package in R software (version 4.3.2; R Foundation for Statistical Computing, Vienna, Austria).

## 3 Results

### 3.1 Baseline Clinical Characteristics

A total of 145 patients underwent attempted LBBAP, of whom 126 (86.9%) achieved successful implantation and 19 (13.1%) experienced unsuccessful implantation. Among the 19 unsuccessful cases, 13 (68.4%) were attributable to inability to adequately penetrate the interventricular septum, whereas 6 (31.6%) achieved deep septal lead deployment but failed to fulfill the predefined electrocardiographic criteria for left bundle branch capture. Baseline characteristics are summarized in Table 1. There were no significant differences between groups in age (77.4 ± 11.2 vs. 76.7 ± 6.8 years; *p* = 0.79), body mass index, or the prevalence of hypertension, dyslipidemia, diabetes mellitus, prior heart failure, or atrial fibrillation. However, the proportion of male patients was significantly higher in the unsuccessful group compared with the successful group (84.2% vs. 57.9%, *p* = 0.02). Electrocardiographically, baseline QRS duration and QRS morphology were similar between the two groups. Baseline left bundle branch block (LBBB) morphology was present in 22 patients (15.2%) overall, including 3 patients (15.8%) in the unsuccessful group and 19 patients (15.1%) in the successful group (*p* = 0.94). In contrast, NIVCD was significantly more frequent in patients with unsuccessful LBBAP (36.8% vs. 11.9%, *p* = 0.004). Echocardiographic evaluation demonstrated notable structural differences. Patients in the unsuccessful group had significantly larger LV end‐diastolic diameter (50.5 ± 4.6 vs. 47.6 ± 5.6 mm, *p* = 0.03), increased IVSd (10.4 ± 1.7 vs. 8.9 ± 1.4 mm, *p* = 0.002), and markedly enlarged RAA (20.1 ± 4.3 vs. 16.4 ± 4.8 cm^2^, *p* = 0.001). LVEF, LVDd, left atrial volume index, and tricuspid regurgitation grade did not differ significantly between groups. Right‐sided lead implantation was also more common in the unsuccessful group (15.8% vs. 3.2%, *p* = 0.01). The proportion of patients fulfilling each predefined electrophysiologic criterion differed markedly between groups. Among successful cases, output‐dependent QRS transition was observed in 72%, V6 RWPT < 75 ms in 81%, V6–V1 interpeak interval ≥ 44 ms in 65%, and concordant intrinsic–paced RWPT in 58%. In contrast, none of these criteria were satisfied in the majority of unsuccessful cases. The detailed distribution of each criterion in both groups is summarized in Table S1.

**Table 1:** Baseline characteristics of the study population.

	Unsuccessful LBBAP	Successful LBBAP	*p*
	*N* = 19	*N* = 126	
Age, years	76.7 ± 6.8	77.4 ± 11.2	0.79
Male, *n* (%)	16 (84.2)	73 (57.9)	0.02
BMI	22.4 ± 3.8	22.3 ± 3.6	0.90
Hypertension, *n* (%)	17 (89.5)	105 (83.3)	0.47
Dyslipidemia, *n* (%)	7 (36.8)	50 (39.7)	0.81
Diabetes mellitus, *n* (%)	6 (31.6)	27 (21.4)	0.43
Prior HF, *n* (%)	6 (31.6)	58 (46.0)	0.23
Paroxysmal AF, *n* (%)	3 (20.0)	40 (33.3)	0.27
Cardiomyopathy			
ICM, *n* (%)	2 (10.5)	10 (7.9)	0.71
NICM, *n* (%)	5 (26.3)	28 (22.3)	0.69
Valvular heart disease, *n* (%)	1 (5.3)	22 (12.5)	0.12
Indication			
1st degree AVB, *n* (%)	2 (10.5)	23 (15.9)	0.27
2nd degree AVB, *n* (%)	8 (42.1)	47 (37.3)	0.16
High or 3rd degree AVB, *n* (%)	10 (52.6)	52 (43.3)	0.68
Laboratory data			
Hemoglobin, g/dL	12.4 ± 2.3	12.7 ± 1.9	0.49
eGFR, mL/min/1.73 m^2^	49.3 ± 17.5	54.8 ± 19.8	0.25
NT‐proBNP, pg/mL	796 (471–1357)	633.5 (299–1509)	0.83
Medications			
β‐blocker, *n* (%)	7 (36.8)	43 (34.1)	0.82
Ca‐blocker, *n* (%)	15 (78.9)	99 (78.6)	0.97
ACE‐I/ARB, *n* (%)	16 (84.2)	95 (75.4)	0.37
Diuretics, *n* (%)	7 (36.8)	49 (38.9)	0.87
Electrocardiographic parameters			
Baseline QRS duration, ms	121.4 ± 25.6	116.6 ± 24.6	0.43
Baseline QRS morphology			
LBBB, *n* (%)	3 (15.8)	19 (15.1)	0.94
RBBB, *n* (%)	4 (21.1)	43 (34.1)	0.24
NIVCD, *n* (%)	7 (36.8)	15 (11.9)	0.004
Echocardiographic parameters			
LVDd, mm	50.5 ± 4.6	47.6 ± 5.6	0.03
LVDs, mm	35.3 ± 6.2	32.7 ± 6.1	0.09
LVEF, %	55.8 ± 11.3	58.7 ± 9.7	0.24
IVSd, mm	10.4 ± 1.7	8.9 ± 1.4	0.002
LAVI	38.0 ± 12.5	40.9 ± 18.9	0.52
TR grade	1.5 ± 0.9	1.3 ± 0.9	0.65
RAA, cm^2^	20.1 ± 4.3	16.4 ± 4.8	0.001
Right‐sided implantation, *n* (%)	3 (15.8)	4 (3.2)	0.01

Abbreviations: ACE‐I, angiotensin‐converting enzyme inhibitor; AF, atrial fibrillation; ARB, angiotensin receptor blocker; AVB, atrioventricular block; BMI, body mass index; eGFR, estimated glomerular filtration rate; HF, heart failure; ICM, ischemic cardiomyopathy; IVSd, interventricular septum thickness; LAVI, left atrial volume index; LBBAP, left bundle branch area pacing; LBBB, left bundle branch block; LVDd, left ventricular end‐diastolic diameter; LVDs, left ventricular systolic diameter; LVEF, left ventricular ejection fraction; NICM, non‐ischemic cardiomyopathy; NIVCD, nonspecific intraventricular conduction delay; NT‐proBNP, N‐terminal pro‐B type natriuretic peptide; RAA, right atrial area; RBBB, right bundle branch block; TR, tricuspid regurgitation.

### 3.2 Predictive Model for Unsuccessful LBBAP

**Figure 2:**
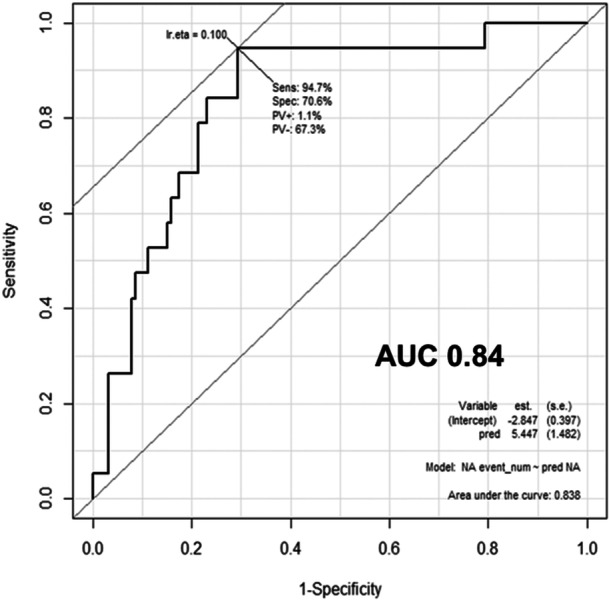
Receiver operating characteristic (ROC) curve of the prediction model for unsuccessful LBBAP. The model showed good discrimination with an AUC of 0.84. The optimal cutoff provided a sensitivity of 94.7% and specificity of 70.6%.

Univariable logistic regression identified several variables significantly associated with unsuccessful LBBAP, including presence of NIVCD, greater IVSd, increased RAA, larger LVDd, male sex, and right‐sided lead implantation (Table 2). In the multivariable model using Firth's penalized likelihood method, three variables remained independent predictors of unsuccessful LBBAP: NIVCD (OR = 4.34, 95% CI = 1.28–14.33; *p* = 0.02), greater IVSd (OR = 1.39 per mm, 95% CI = 1.02–1.96; *p* = 0.03), and larger RAA (OR = 1.12 per cm^2^, 95% CI = 1.02–1.25; *p* = 0.01). Right‐sided implantation was significantly associated with unsuccessful LBBAP in univariable analysis but was not retained in the final multivariable model. Patients undergoing right‐sided implantation tended to have larger right atrial area compared with those undergoing left‐sided implantation (18.7 ± 2.9 vs. 16.2 ± 5.0 cm^2^, *p* = 0.08). The predictive model demonstrated strong discriminative performance, with an AUC of 0.84 (Figure 2). The optimal cutoff value, determined using the Youden index, provided a sensitivity of 94.7% and a specificity of 70.6%, with a negative predictive value of 97.3%. We developed a simplified point‐based risk score derived from the *β* coefficients of the final multivariable logistic regression model. A simplified point‐based score (0–4 points), derived from regression coefficients (NIVCD = 2 points; IVSd > 10 mm = 1 point; RAA > 18 cm^2^ = 1 point), demonstrated comparable discriminative performance (AUC = 0.82) and is presented in Figure 1.

**Table 2:** Univariable and multivariable logistic regression analyses for predictors of unsuccessful LBBAP.

Variables	Odds ratio (OR)	95% CI	*p*	OR	95% CI	*p*
Age	0.99	0.95–1.04	0.68			
Man	3.43	1.14–13.61	0.03			
NIVCD	4.32	1.46–12.34	0.009	4.34	1.28–14.33	0.02
Right‐sided implantation	5.77	1.19–25.95	0.03			
IVSd	1.65	1.24–2.26	0.001	1.39	1.02–1.96	0.03
LVDd	1.09	1.00–1.18	0.038			
RAA	1.14	1.04–1.25	0.004	1.12	1.02–1.25	0.01

Abbreviations: IVSd, interventricular septum thickness; LBBAP, left bundle branch area pacing; LVDd, left ventricular end‐diastolic diameter; NIVCD, nonspecific intraventricular conduction delay; RAA, right atrial area.

### 3.3 Validation of the Prediction Model

**Figure 3:**
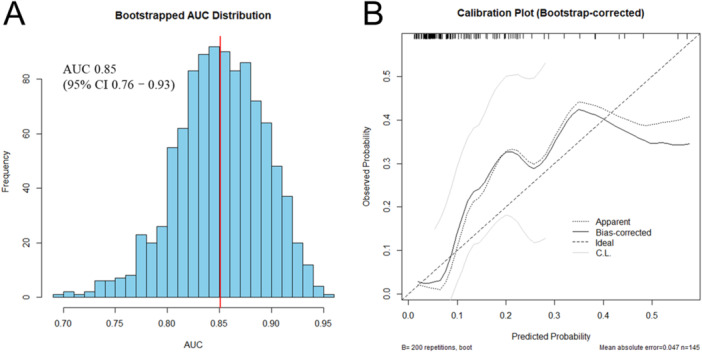
(A) Bootstrap‐validated receiver operating characteristic (ROC) curve for the prediction model. The ROC curve shows the discrimination of the multivariable model after 1000 bootstrap resamples. The optimism‐corrected AUC was 0.85 (95% CI = 0.76–0.93), indicating preserved predictive accuracy after internal validation. (B) Calibration plot of the prediction model after bootstrap correction. The bias‐corrected calibration curve demonstrates close alignment between predicted and observed probabilities. The curve tracks the ideal 45° line with minimal deviation, and the mean absolute calibration error was 0.047, indicating good calibration performance.

Internal validation using 1000 bootstrap resamples demonstrated good model stability. The optimism‐corrected AUC was 0.85 (95% CI = 0.76–0.93), indicating preserved discriminative performance after correction for potential overfitting (Figure 3A).

Calibration analysis using bootstrap‐corrected curves showed close agreement between predicted and observed probabilities across the full risk range. The bias‐corrected calibration curve closely followed the ideal 45° line, with only minor deviations at higher predicted probabilities. The mean absolute calibration error was 0.047, confirming satisfactory model calibration (Figure 3B).

### 3.4 Nomogram for Individualized Risk Estimation

**Figure 4:**
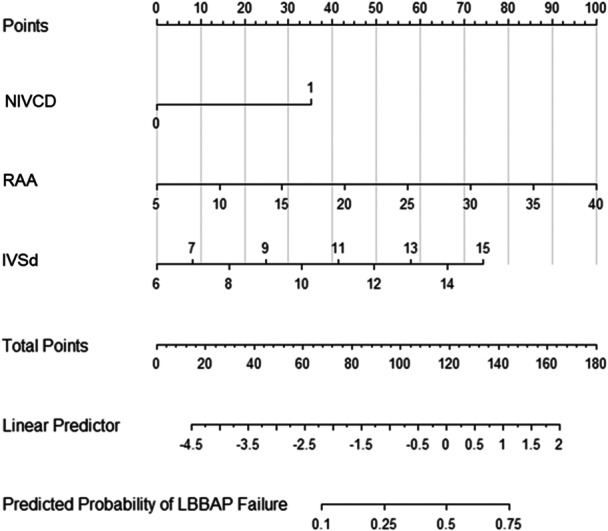
Nomogram for predicting unsuccessful LBBAP. The nomogram integrates three independent predictors—NIVCD, RAA, and IVSd—derived from the final multivariable model. Summing the point values corresponding to each variable yields an individualized estimate of the probability of unsuccessful LBBAP.

**Figure 5:**
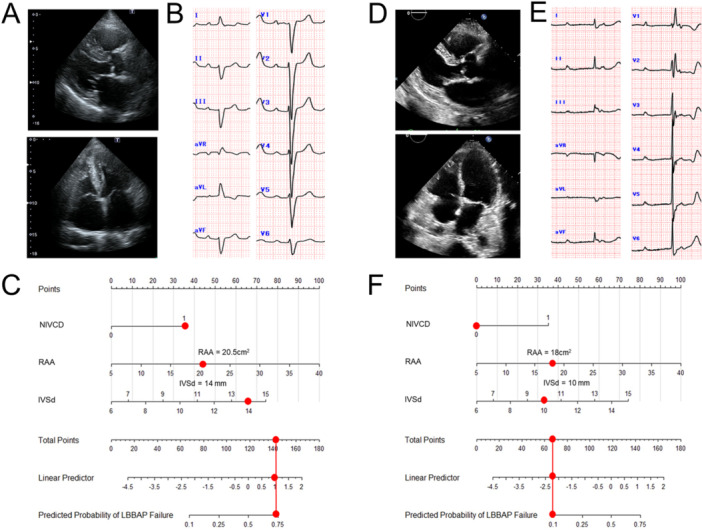
Representative application of the nomogram for predicting unsuccessful LBBAP. Panels (A–C) High‐risk case. (A) Echocardiography showing septal thickening and right atrial enlargement. (B) Baseline ECG demonstrating nonspecific intraventricular conduction delay. (C) Nomogram application (RAA = 20.5 cm^2^, IVSd = 14 mm, NIVCD present) yielding an estimated 75% probability of LBBAP failure. Panels (D–F) Low‐risk case. (D) Echocardiography showing normal septal thickness and right atrial size. (E) Baseline ECG without NIVCD. (F) Nomogram application (RAA = 18 cm^2^, IVSd = 10 mm, NIVCD absent) yielding an estimated 10% probability of LBBAP failure.

To facilitate individualized risk assessment, a nomogram was developed based on the final multivariable logistic regression model (Figure 4). The nomogram incorporates the three independent predictors—NIVCD, RAA, and IVSd—with each variable assigned a weighted point value proportional to its regression coefficient. By summing the points corresponding to a patient's clinical profile and projecting the total onto the probability scale, clinicians can estimate the individualized risk of unsuccessful LBBAP. This graphical tool enables intuitive bedside risk calculation and may aid in pre‐procedural planning and decision‐making. Representative high‐ and low‐risk cases illustrating practical application of the nomogram are presented in Figure 5.

## 4 Discussion

In the present study, including patients undergoing attempted LBBAP, we identified three independent predictors of unsuccessful implantation: presence of NIVCD, increased IVSd, and enlarged right atrial area. Based on these parameters, we developed a multivariable logistic regression model with strong discriminative performance and good calibration, which was further supported by machine learning. Using this model, we constructed a nomogram that enables individualized prediction of LBBAP implantation failure.

Previous investigations have described several clinical or anatomical factors associated with technical difficulty in LBBAP, such as left ventricular dilation, non‐left bundle branch block conduction patterns, septal hypertrophy, and heart failure [11, 12, 13]. However, these reports were largely descriptive, and no prior study has provided a quantitative, validated tool to estimate the likelihood of LBBAP failure on an individual basis. To our knowledge, this study is the first to propose a standardized, internally validated prediction model and nomogram for unsuccessful LBBAP.

NIVCD was the strongest independent predictor of unsuccessful implantation in our cohort. NIVCD represents a diffuse delay in intraventricular conduction rather than isolated bundle branch block and is often associated with underlying structural heart disease or myocardial fibrosis [22, 23]. In such patients, ventricular activation is already prolonged and heterogeneous at baseline, which may alter septal activation patterns and make it more difficult to fulfill the currently accepted electrocardiographic criteria for successful LBBAP. Even when the pacing lead is positioned close to the left bundle branch area, these diffuse conduction abnormalities may blunt the expected changes in QRS morphology or timing, thereby reducing the likelihood that the implantation will be judged successful according to current criteria. Importantly, NIVCD may influence not only the technical feasibility of achieving left bundle capture but also the ability to confirm capture using electrocardiographic criteria. In our cohort, among unsuccessful cases with baseline NIVCD (*n* = 7), five patients (71.4%) achieved deep septal lead deployment but failed to fulfill electrocardiographic criteria for LBB capture. This observation suggests that in the presence of diffuse intraventricular conduction delay, currently accepted ECG‐based definitions of LBB capture may underestimate successful conduction system engagement. Therefore, NIVCD may partly reflect diagnostic limitations rather than purely technical failure. NIVCD has been associated with reduced response to various pacing modalities, including conventional right ventricular pacing and cardiac resynchronization therapy. Therefore, NIVCD may reflect a diffuse myocardial conduction substrate that limits effective resynchronization irrespective of the pacing strategy employed. Increased IVSd was also independently associated with implantation failure. A thickened interventricular septum, commonly seen in hypertensive heart disease or hypertrophic cardiomyopathy, poses serious mechanical challenges during LBBAP. The pacing lead may not penetrate deeply enough to reach the left bundle branch area, particularly when the septal myocardium is markedly thick or fibrotic. Septal thickness demonstrated moderate discriminative ability for predicting unsuccessful LBBAP (AUC = 0.75). Although an IVSd value of approximately 10 mm provided balanced sensitivity and specificity in our cohort, this threshold corresponds to the upper limit of normal and therefore may not represent a clinically distinct pathological cutoff. A higher threshold of 11 mm—commonly used to define left ventricular hypertrophy—was associated with higher specificity but substantially lower sensitivity. These findings suggest that IVSd should be interpreted as a continuous marker of procedural risk rather than a strict binary determinant. Accordingly, individualized risk estimation using the nomogram may provide greater clinical utility than reliance on a single threshold value. Our study additionally identified RAA enlargement as a novel anatomical predictor of unsuccessful LBBAP. Beyond reflecting chronic right‐sided pressure elevation or atrial remodeling, right atrial enlargement may also contribute to failure through a mechanical mechanism. A markedly enlarged right atrium can prevent the delivery catheter from sufficiently entering the RV, resulting in inadequate backup support. Adequate backup force is essential for deep septal penetration and for maintaining stable perpendicular alignment between the sheath and the interventricular septum. When backup is insufficient, the lead may fail to advance through hypertrophied or remodeled septal tissue, thereby increasing the likelihood of unsuccessful implantation. Although right‐sided implantation was significantly associated with unsuccessful LBBAP in univariable analysis, it was not included in the final multivariable model. Given the limited number of failure events, the model was intentionally restricted to a small number of clinically relevant variables to minimize the risk of overfitting. In our cohort, patients undergoing right‐sided implantation tended to have larger right atrial area compared with those undergoing left‐sided implantation, suggesting partial overlap between implantation side and anatomical characteristics. To avoid potential collinearity and maintain model stability, RAA was selected as a more direct anatomical variable in the final parsimonious model. Furthermore, previous studies have demonstrated that right‐sided LBBP using a lumen‐less lead is technically feasible and associated with high procedural success, albeit with longer fluoroscopy times [24]. These observations suggest that implantation side alone may not universally determine procedural success.

Our findings are consistent with prior evidence. In a large multicenter Japanese cohort, Kato et al. reported that LBBAP success was markedly reduced in patients with IVCD (50%), and IVCD emerged as the strongest independent predictor of procedural failure [11]. Similarly, septal hypertrophy was also an independent predictor in their analysis, with most failures caused by the inability to advance the lead deeply into the thickened septum. The distribution of failure mechanisms in our cohort was consistent with these multicenter observations, in which mechanical septal penetration failure accounted for the majority of unsuccessful cases. This concordance supports the reproducibility of the underlying mechanisms of LBBAP failure across different clinical settings. Furthermore, severe tricuspid regurgitation was associated with failure, likely due to right‐sided chamber dilation impairing sheath support; this supports the mechanical rationale behind our finding that right atrial enlargement predicts unsuccessful implantation.

The prediction model and nomogram developed in this study enable intuitive, individualized estimation of the likelihood of LBBAP failure. Such pre‐procedural risk stratification may assist clinicians in anticipating procedural difficulty and facilitating informed decision‐making and patient counseling. Furthermore, this framework may provide a basis for future studies to perform risk adjustment when evaluating conduction system pacing strategies.

## 5 Limitations

Several limitations warrant consideration. First, the overall sample size was modest, and the number of unsuccessful implantation events was limited (*n* = 19). Although Firth's penalized likelihood method and bootstrap validation were applied to mitigate small‐sample bias, residual uncertainty cannot be completely excluded. Second, this study was conducted at a single high‐volume center with substantial experience in conduction system pacing. All procedures were performed by operators who had surpassed the early learning phase of LBBAP implantation. Therefore, the reproducibility of these findings in lower‐volume centers or during the initial learning curve remains uncertain. Third, patients with an indication for cardiac resynchronization therapy were excluded, and most participants had preserved LVEF (> 50%). Accordingly, the applicability of our prediction model to patients with reduced ejection fraction or more advanced heart failure cannot be determined. Fourth, all procedures were performed using a lumen‐less pacing lead in a high‐volume center with substantial experience in conduction system pacing. Therefore, the generalizability of our findings to stylet‐driven pacing leads or newer delivery systems remains uncertain and warrants further investigation. Finally, the study relied on currently accepted electrocardiographic criteria for defining successful LBB capture. Accordingly, failure in this study was defined based on these ECG criteria; therefore, the model predicts failure as operationally defined by ECG endpoints rather than confirmed absence of conduction system engagement. In patients with diffuse conduction delay, such as those with NIVCD, these criteria may underestimate successful conduction system engagement. Future studies incorporating alternative diagnostic approaches or imaging‐based validation may further refine the assessment of conduction system capture.

## 6 Conclusions

NIVCD, septal hypertrophy, and right atrial enlargement were independent predictors of unsuccessful LBBAP. The resulting validated prediction model and nomogram provide individualized risk estimation and may assist in pre‐procedural planning and patient counseling.

## Funding

The authors have nothing to report.

## Ethics Statement

The study protocol was approved by the Institutional Ethics Committee of the Graduate School of Biomedical Science at Hiroshima University.

## Consent

The institutional ethics committee determined that written informed consent was not required due to the retrospective observational nature of the study.

## Conflicts of Interest

The authors declare no conflicts of interest.

## Supporting information


Supporting File:

## Data Availability

The data that support the findings of this study are available from the corresponding author upon reasonable request.
