# Supplementary material for: Clinical Prediction Model for Unsuccessful Left Bundle Branch Area Pacing
Source: J Cardiovasc Electrophysiol. 2026 Apr 11;37(6):1211–9. doi: 10.1111/jce.70339 (PMC13269862; doi:10.1111/jce.70339)
Supplement: Supplementary file 1 — Supporting File: [file JCE-37-1211-s001.docx]

**Supplementary Figure 1**. Receiver operating characteristic curve of the simplified point-based risk score


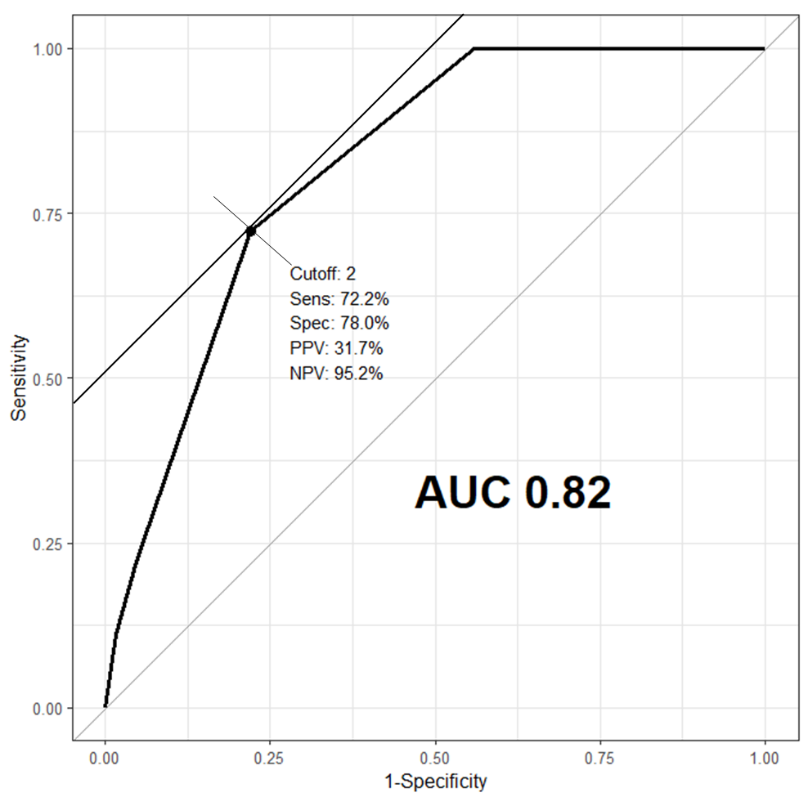


Receiver operating characteristic (ROC) curve demonstrating the discriminative performance of the simplified point-based risk score (0–4 points) for predicting unsuccessful left bundle branch area pacing (LBBAP). The area under the curve (AUC) was 0.82. The optimal cutoff value (≥2 points), determined using the Youden index, yielded a sensitivity of 72% and specificity of 78%. The diagonal line represents the line of no discrimination.

**Supplementary Table 1**. Distribution of predefined electrophysiologic criteria for successful LBBAP in the successful and unsuccessful groups

| Electrophysiologic criterion | Unsuccessful LBBAP (n = 19) | Successful LBBAP (n = 126) | P value |
| --- | --- | --- | --- |
| Output-dependent QRS transition | 0 (0%) | 74 (58.7) | <0.001 |
| V6 RWPT < 75 ms | 0 (0%) | 114 (91.3) | <0.001 |
| V6–V1 interpeak interval > 44 ms | 0 (0%) | 55 (43.6) | <0.001 |
| Intrinsic–paced V6 RWPT concordance | 0 (0%) | 50 (39.6) | <0.001 |

LBBAP, left bundle branch area pacing; RWPT, R wave peak time
